# MAP2K4 interacts with Vimentin to activate the PI3K/AKT pathway and promotes breast cancer pathogenesis

**DOI:** 10.18632/aging.102485

**Published:** 2019-11-25

**Authors:** Shu Liu, Juan Huang, Yewei Zhang, Yiyi Liu, Shi Zuo, Rong Li

**Affiliations:** 1 Southern Medical University, Nanfang Hospital, Department of Oncology, Guangzhou 510515, Guangdong, P. R. China; 2 Guizhou Maternity and Child Health Hospital, Guiyang 550003, Guizhou, P. R. China; 3 Cancer Center, Integrated Hospital of Traditional Chinese Medicine, Southern Medical University, Guangzhou 510310, Guangdong, P. R. China; 4 Department of General Surgery, The Affiliated Hospital of Guizhou Medical University, Guiyang 550001, Guizhou, P. R. China

**Keywords:** MAP2K4, breast cancer, PI3K/AKT pathway, Vimentin

## Abstract

Mitogen-activated protein kinase kinase 4 (MAP2K4) is a member of the mitogen-activated protein kinase (MAPK) activator family. MAPK signaling plays a significant role in cell proliferation, differentiation, transcriptional regulation, and development. However, specific function and mechanism of MAP2K4 in breast cancer have not been clarified. According to our study, overexpressed MAP2K4 in breast cancer cells increased proliferation, migration, and invasion in vivo and in vitro, while MAP2K4 knockdown restored the effects. Subsequent mechanistic analyses demonstrated that MAP2K4 promoted cell proliferation, migration, and invasion by activating phosphoinositide-3-kinase (PI3K)/AKT signaling, the downstream proteins, c-JUN, the G1/S cell cycle, and the epithelial-to-mesenchymal transition (EMT). Meanwhile, MAP2K4 interacted with Vimentin and further propagated the malignant phenotype. Furthermore, patients with high MAP2K4 and Vimentin expression levels had poorer overall survival rates than those with low expression levels of both proteins. Our studies demonstrated that MAP2K4 has the potential to serve as an oncogene in breast cancer and it activates the phosphorylated PI3K/AKT signaling pathway to activate downstream cycle-associated proteins and EMT signals while interacting with Vimentin to promote breast cancer cells proliferation, migration, and invasion. In our study, MAP2K4 and Vimentin co-expression is confirmed to be an unfavorable factor in breast cancer.

## INTRODUCTION

Breast cancer is one of the most common malignancies in women worldwide [1]. In China, breast cancer alone accounted for 15% of all new cancers in women in 2015 [2], which lead to substantial mental and economic burdens. Breast cancer is a heterogeneous disease derived from mammary gland epithelial tissue, which is related to age, estrogen, family history, gene susceptibility, and other factors [3, 4]. At present, a variety of gene mutations and abnormal proteins expression are involved in the pathogenesis of breast cancer. Thus, new therapeutic targets against breast cancer could be identified through the study of breast cancer-related genes and the pathogenesis of this disease.

Mitogen-activated protein kinase kinase 4 (MAP2K4, also known as MKK4, MEK4, or SEK1) is a member of the mitogen-activated protein kinase activator family [5]. MAP2K4 is located downstream of the MAPK pathway, which is a signaling cascade of three kinases, including MAPKKK that phosphorylates serine and threonine residues of MAPKK to activate MAPK signaling, that further phosphorylates MAP2K4. MAP2K4 then phosphorylates and activates JNK, MAPK, and p38 MAPK, regulates inflammation and cell proliferation, and plays a vital role in tumor development [6]. It has been reported that the JNK, MAPK, and p38 MAPK pathways are associated with tumor suppression [7]. However, there are additional reports indicating that MAP2K4 and JNK can participate in the promotion of tumorigenesis [8], suggesting that the molecular mechanisms of this signaling pathway in tumor progression is very complicated. Many studies have found that mutations or abnormal MAP2K4 expression are involved in the pathogenesis of various tumors. It has been reported that MAP2K4, which is highly expressed in osteosarcoma, inhibits cell proliferation and migration by activating the JNK/p38 signaling pathway [9]. In addition, MAP2K4 signaling was shown to be involved in the EMT process of tumors. In ovarian cancer, MAP2K4 overexpression inhibits NF-κB phosphorylation and up-regulates E-cadherin expression in epithelial cells to inhibit EMT in ovarian cancer [10]. Studies of lung adenocarcinoma found that MAP2K4 overexpression inhibits tumor cell invasion by down-regulating PPARγ2 [11], showing that MAP2K4 acts as a tumor suppressor gene in tumor progression. However, other investigations have confirmed that MAP2K4 acts as an oncogene to promote the development of tumors. Studies in prostate cancer demonstrated that overexpressed MAP2K4 promotes prostate cancer metastasis through HSP27 upregulation, which mediates MMP-2 upregulation [12]. In addition, MAP2K4 activates p38 protein to induce the transformation of prostate cancer epithelial cells into mesenchymal cells, which results in distant tumor cell metastases. Although MAP2K4 was reported in some tumors, its specific function and mechanism in breast cancer have not been clarified, and further investigation is needed.

In a breast cancer pilot study, MAP2K4 was recognized to be a candidate tumor suppressor [13, 14]. Conversely, Wang et al. [15] found that MAP2K4 could act as an oncogene because it can help promote proliferation and inhibit apoptosis of breast cancer cells. Due to the controversial evidence regarding MAP2K4, we previously investigated the expression and clinical value of MAP2K4 in invasive breast cancer [16]. The results showed that MAP2K4 is an independent predictor of outcome. Here, we further explored MAP2K4 biological functions in the regulation of breast cancer signaling pathways. On this topic, we confirmed a new mechanism for MAP2K4 in breast cancer. Different from previous ovarian cancer and lung adenocarcinoma MAP2K4 studies, we confirmed the ability of MAP2K4 to promote breast cancer cells proliferation, invasion, and migration through a series of functional in vitro and in vivo experiments, demonstrating an oncogenic role for MAP2K4 in breast cancer. Regarding the mechanism of action, our study also was the first to show that MAP2K4 overexpression activates the PI3K/AKT pathway and interacts with Vimentin in breast cancer cells. From the above results, we showed that MAP2K4 could have an oncogenic role to promote the occurrence and development of breast cancer.

## RESULTS

### MAP2K4 overexpression promotes cell proliferation, migration, and invasion in breast cancer cells

**Figure 1:**
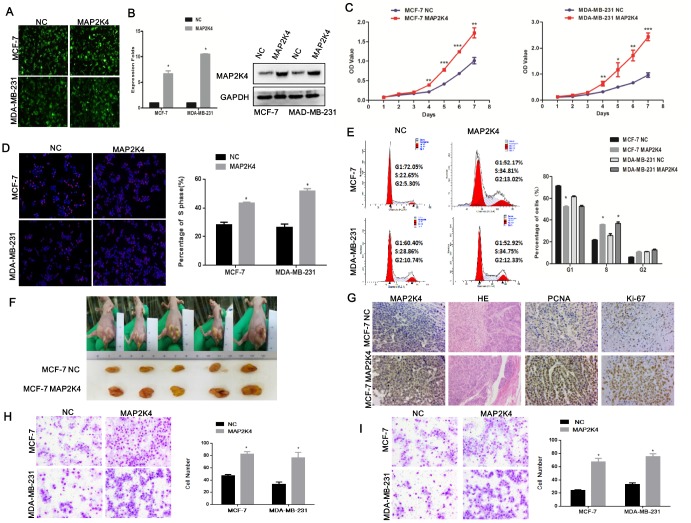
**Overexpression of MAP2K4 promotes cell proliferation, migration, and invasion of breast cancer cells.** (**A**) MCF-7 and MDA-MB-231 cells were transfected with Lv-MAP2K4 and LEV. Green fluorescent protein (GFP) expression was used to monitor the transfection efficiency. (**B**) MAP2K4 expression measured with qRT-PCR and Western blot following Lv-MAP2K4 overexpression, normalized to GAPDH. Mean ± SD,*P<0.05. MTT assays (**C**), EdU assays (**D**), and FCM (**E**) in MCF-7-Lv-MAP2K4 and MDA-MB-231-Lv-MAP2K4 cells. Mean ± SD *P<0.05; **P<0.01; ***P<0.001. (**F**) The in vivo MAP2K4 effect was evaluated in xenograft mouse models bearing tumors originating from MCF-7 cells, n=5/group. (**G**) Representative H&E stained sections with Ki-67 and PCNA IHC staining of primary tumor tissues are shown. Transwell (**H**) and Boyden assays (**I**) were performed to measure the effect on invasion and migration in MCF-7-Lv-MAP2K4 and MDA-MB-231-Lv-MAP2K4 cells. Mean ±SD, *P<0.05; **P<0.01.

To investigate the biological role of MA2PK4 in breast cancer cells, lentivirus-carrying MAP2K4 cDNA was infected into MCF-7 and MDA-MB-231 cells (Figure 1A). Real-time quantitative RT-PCR(qRT-PCR) and Western blot respectively verified higher MAP2K4 mRNA and protein expression levels in MCF-7 and MDB-MA-231 cells than in their respective empty vector control cells (Figure 1B).

Subsequently, we examined the effect of MAP2K4 expression on MCF-7 and MDA-MB-231 cell growth using the MTT assay (Figure 1C), EdU staining (Figure 1D), and cell cycle analysis. We found that overexpressed MAP2K4 markedly promoted cell growth and the G1 to S cell-cycle transition in MCF-7 and MDA-MB-231 cells, *in vitro* (Figure 1E). Finally, we confirmed the effect of MAP2K4 on cell proliferation *in vivo* by subcutaneously inoculating MAP2K4-overexpressing MCF-7 and MDA-MB-231 cells into nude mice. The average weight and volume of the tumors were noticeably increased in MAP2K4-overexpressing xenograft mice compared with mock xenograft mice (Figure 1F). Furthermore, proliferating cell nuclear antigen (PCNA) and Ki-67 expression as cellular markers for proliferation were ubiquitously higher in tumor tissues with MAP2K4 overexpression compared with those of the control tissues (Figure 1G).

Next, Transwell (Figure 1H) and Boyden (Figure 1I) assays were performed to evaluate the effects of MAP2K4 on metastatic migration and invasion, *in vitro*. Consistent with the cell proliferation assays above, MAP2K4-overexpressing cell lines promoted invasive and migratory abilities compared with control cells. The results demonstrated that overexpressed MAP2K4 markedly stimulated cell migration and invasion in breast cancer cells.

### Silencing of MAP2K4 expression inhibits breast cancer cell proliferation, migration, and invasion

**Figure 2:**
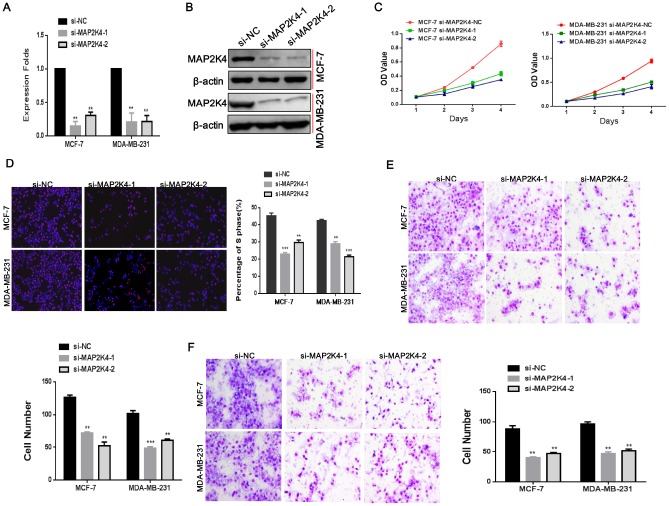
**Silencing of MAP2K4 inhibits proliferation, migration, and invasion of breast cancer cells.** (**A**) and (**B**),Two siRNA sequences were transfected to knockdown MAP2K4 expression in MCF-7 and MDA-MB-231 cells. MAP2K4 expression was detected with qRT-PCR and Western blot in MAP2K4-silenced breast cancer cells, normalized to GAPDH or β-actin. Mean ± SD,**P<0.01. Cell proliferation, migration, and invasion were measured by MTT assays (**C**), EdU assays (**D**), Transwell (**E**) and Boyden assays (**F**). Mean ± SD, *P<0.05; **P<0.01; ***P<0.001.

To confirm that MAP2K4 does affect the proliferation, migration, and invasion of breast cancer cells, *in vitro*, we transiently transfected two siRNA sequences to knockdown MAP2K4 expression in breast cancer cells. We found effective MAP2K4 silencing as identified by qRT-PCR and Western blot (Figure 2A, 2B) in MCF-7 and MDA-MB-231 cells compared with the corresponding control cells without siRNA sequences.

In line with our previous studies, we found that siMAP2K4s could notably reverse cell growth stimulation, the G1 to S cell-cycle transition, migration, and invasion of MCF-7 and MDA-MB-231 cells by MTT (Figure 2C), EdU staining (Figure 2D), Transwell (Figure 2E) and Boyden assays (Figure 2F) compared with the control parental lines. These results validated that MAP2K4 might serve as an oncogene in breast cancer.

### MAP2K4 activates PI3K/AKT and its downstream cell cycle and EMT signals

**Figure 3:**
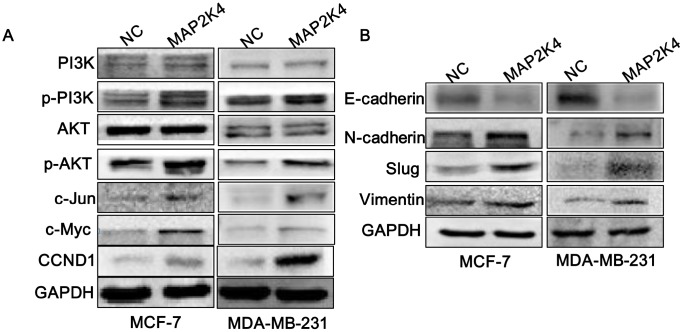
**MAP2K4 activates PI3K/AKT and its downstream cell cycle and EMT signals. Western blot analysis of proteins in response to overexpressed MAP2K4 in MCF-7 and MDA-MB-231 cells.** (**A**) PI3K, p-PI3K, AKT, p-AKT, c-JUN, c-Myc and CCND1 protein sequences, (**B**) the invasion and migration associated proteins, E-cadherin, N-cadherin, Slug, and Vimentin were measured.

To explore the specific molecular mechanisms by which MAP2K4 functions as a breast cancer oncogene, we conducted Western blot analyses to analyze key regulators of the cell cycle and found that MAP2K4 overexpression upregulated cell cycle-related genes including p-PI3K, p-AKT, c-JUN, c-Myc, and Cyclin D1(CCND1) in both MCF-7 and MDA-MB-231 cells. These results suggest that MAP2K4 increased cell growth by activating phosphoinositide-3-kinase (PI3K)/AKT as well as downstream c-JUN and G1/S cell-cycle transition signaling (Figure 3A).

Based on the invasion and migration assay results, we examined EMT signals in MAP2K4-overexpressing breast cancer cells and observed that N-cadherin, Vimentin, and Slug were significantly elevated while E-cadherin was decreased, as we expected (Figure 3B).

### Suppressing p-PI3K reduces cell proliferation, migration, and invasion of MAP2K4-overexpressing breast cancer cells

**Figure 4:**
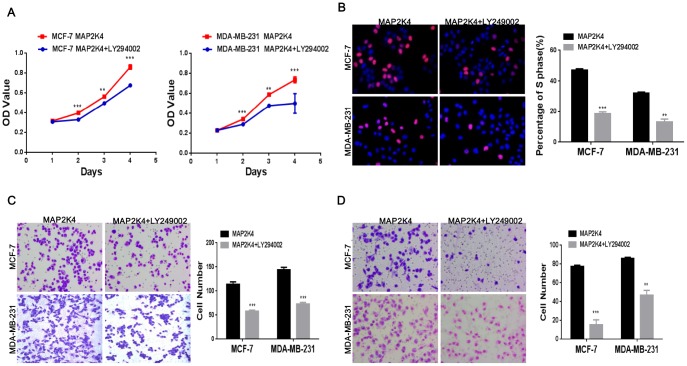
**Inhibiting p-PI3K expression reduces cell proliferation, migration, and invasion in MAP2K4-overexpressing breast cancer cells.** MTT assays (**A**), EdU incorporation assay (**B**) and Transwell (**C**) and Boyden assays (**D**) of breast cancer cells were conducted after inhibiting p-PI3K expression. Mean ± SD, *P<0.05; **P<0.01; ***P<0.001.

In the subsequent study, we suppressed p-PI3K expression using the LY294002 inhibitor (a broad-spectrum inhibitor of PI3K) to further define the specific pathway that MAP2K4 regulates cellular function. Western blots supported its effectively inhibition. Consequently, we explored that suppressing p-PI3K observably weaken cell growth, the G1 to S cell-cycle transition, migration, and invasion in MAP2K4-overexpressing MCF-7 and MDA-MB-231 cells using the MTT assay (Figure 4A), EdU staining (Figure 4B), and the Transwell (Figure 4C) and Boyden assays (Figure 4D) compared with controls. These data showed that p-PI3K plays a critical role in the regulation of cellular function by MAP2K4 in breast cancer.

Taken together, the results strongly revealed that MAP2K4 promotes cell growth, migration, and invasion via the PI3K/AKT signal pathway.

### MAP2K4 interacts with Vimentin

**Figure 5:**
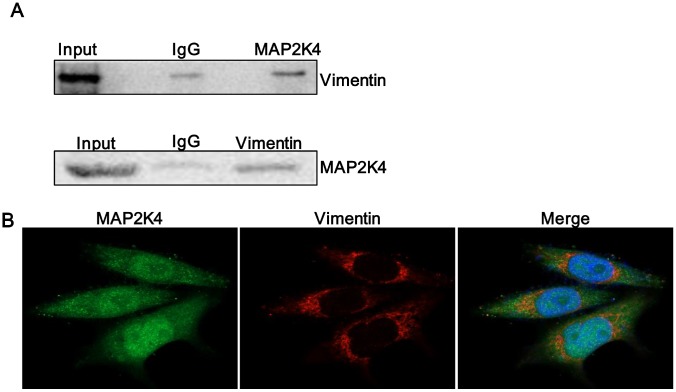
**MAP2K4 interacts with Vimentin.** (**A**) A Co-immunoprecipitation assay was performed to identify if Vimentin coprecipitated with MAP2K4. (**B**) MAP2K4-overexpressing MCF-7 cells were subjected to Vimentin immunofluorescent staining to reveal MAP2K4 and Vimentin colocalization.

The previous study indicated that a non-invasive breast cancer cell line acquired increased motility and invasiveness upon Vimentin overexpression while the characteristics were down-regulated by silencing of Vimentin in an invasive tumor cell line that constitutively expressed [17]. Through Mass Spectrometry assay in our study, Vimentin was predicted to interact with MAP2K4. Thus, we subsequently conducted Co-immunoprecipitation (CoIP) assay (Figure 5A) to further identify Vimentin coprecipitated with MAP2K4, meanwhile Immuno-fluorescent (IF) staining (Figure 5B) confirmed a common cytoplasm colocalization between MAP2K4 and Vimentin in MCF-7 cells.

Vimentin is an EMT-related molecule and high expression in tumors is an important indicator of tumor invasion and metastasis. MAP2K4 can up-regulate Vimentin and interact with Vimentin. Based on these findings, we supposed that MAP2K4 promotes migration and invasion of breast cancer cells by interacting with Vimentin. The experimental results provide insight into the biological significance of the protein-protein interaction of MAP2K4.

### Suppressing Vimentin reduces cell proliferation, migration, and invasion in MAP2K4-overexpressing breast cancer cells

To identify if Vimentin was functional, we introduced a siRNA to inhibit the expression of Vimentin in MAP2K4-overexpressing MCF-7 and MDA-MB-231 cells and observed that Vimentin expression was significantly lower in the inhibitor-treated cells than that in the control cells.

**Figure 6:**
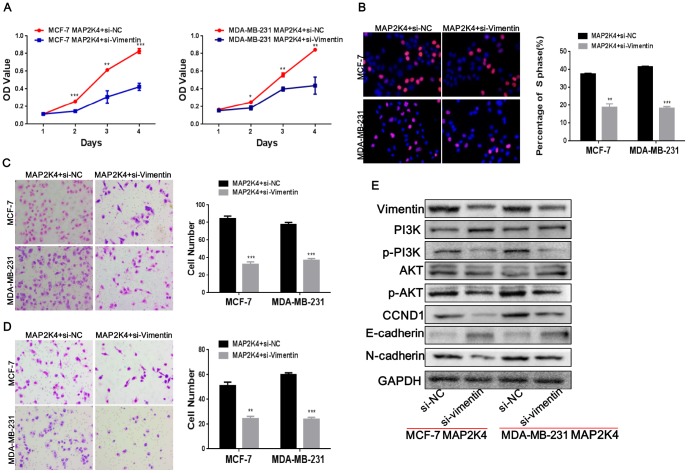
**Vimentin suppression reduces cell proliferation, migration, and invasion in MAP2K4-overexpressing breast cancer cells.** MCF-7-Lv-MAP2K4 and MDA-MB-231-Lv-MAP2K4 cells were transfected with siRNA to inhibit Vimentin expression. Cell growth and cell cycle were measured using the MTT (**A**) and EdU assays (**B**). Invasion and migration were measured with the Transwell and Boyden Chamber assays (**C**, **D**). Mean ± SD, *P<0.05; **P<0.01; ***P<0.001. (**E**) Knocking down Vimentin suppressed PI3K/AKT and further modulated its downstream cell cycle and EMT-associated factors in MAP2K4-overexpressing breast cancer cells.

Transiently transfecting with siRNA to inhibit Vimentin expression in MAP2K4-overexpressing breast cancer cells reduced cell proliferation by MTT (Figure 6A) and EdU incorporation assays (Figure 6B) as well as inhibited G1 to S cell-cycle transition. For invasion and migration analysis, Vimentin-suppressing breast cancer cells and control parental lines were cultured in Transwells (Figure 6C) and Boyden Chambers (Figure 6D). We found that Vimentin inhibition simultaneously reduced invasive and migratory abilities compared with that of the controls. Finally, we observed that knocking down Vimentin suppressed p-PI3K, p-AKT, c-JUN, c-Myc, and Cyclin D1(CCND1), N-cadherin and restored E-cadherin expression in MAP2K4-overexpressed breast cancer cells (Figure 6E).

### MAP2K4 and Vimentin co-expression is an unfavorable factor in breast cancer

**Figure 7:**
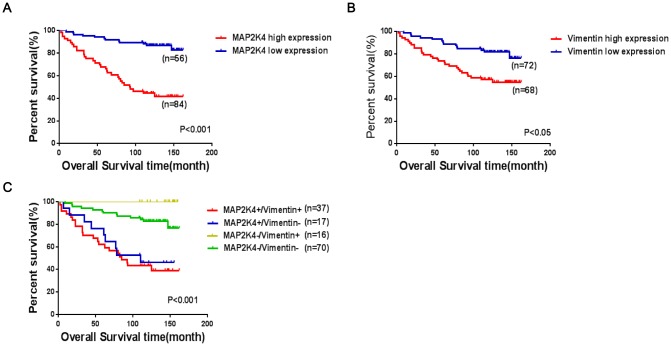
**Co-expression of MAP2K4 and Vimentin is an unfavorable factor in breast cancer patient prognoses.** (**A**–**C**) The log-rank test was used to calculate P-values.

To investigate clinicopathologic correlation, immunohistochemical (IHC) staining was performed to examine MAP2K4 and Vimentin expression patterns in 140 breast cancer specimens concerning several standard clinicopathologic parameters. During a 2-162 month follow-up, 38 patients suffered a recurrence or death with an average survival time of 109.5 months (within the follow-up period), 86.2 months in the MAP2K4 high-expressing group (within the follow-up period), and 125 months in the MAP2K4 low-expressing group (during the follow-up period). In summary, Patients with higher MAP2K4 expression had shorter overall survival time (Figure 7A) (P < 0.001). Meanwhile, we observed that patients with high Vimentin expression had worse prognoses than those with low expression (Figure 7B) (P < 0.05).

The Spearman’s test showed that MAP2K4 expression was positively correlated with Vimentin expression in the breast cancer patients (γ=0.753, P<0.001) (Table 1).

**Table 1:** Correlation between MAP2K4 and Vimentin expression.

**Vimentin**	**n**	**MAP2K4(%)**		**r**	**P**
		**High**	**Low**		
High	54	37(68.5%)	17(31.5%)	0.753	<0.001
Low	86	16(18.6%)	70(81.4%)		

As shown in Table 2, MAP2K4 and Vimentin co-expression levels were positively correlated with the pathologic grade, tumor size, lymph node metastasis presence, and invasion (P<0.001). To further verify the prognostic value of MAP2K4 and Vimentin co-expression in breast cancer patient tissue, we divided 140 cases into four groups based on MAP2K4 and Vimentin expression patterns: (1) MAP2K4 high expression/Vimentin high expression (HH); (2) MAP2K4 high expression/Vimentin low expression (HL); (3) MAP2K4 low expression/Vimentin high expression (LH); and (4) MAP2K4 low expression/Vimentin low expression (LL). Survival curves were constructed using the Kaplan-Meier method and analyzed with the log-rank test. According to the 140 cases with prognostic information, MAP2K4 and Vimentin co-expression levels markedly correlated with overall survival. Patients with high MAP2K4 and Vimentin expression showed the worst prognoses (Mean average survival time 82.3months) compared with those with low expression of MAP2K4 and vimentin or low expression of one or the other (P<0.001). These results indicated MAP2K4 and Vimentin co-expression to be a prognostic predictor of patient survival.

**Table 2:** Correlation between the clinicopathologic characteristics and MAP2K4 and Vimentin co-expression patterns in breast cancer.

**Variables**	**Vimentin**	**MAP2K4&Vimentin(%)**		
		**n**	**high**	**low**	**P**
Clinical grade					
I-II	high	60	45(75.0%)	15(25.0%)	
	low	69	3(4.3%)	66(95.7%)	<0.001
III-IV	high	7	7(100.0%)	0(0.0%)	
	low	1	0(0.0%)	1(100.0%)	0.005
Tumor size					
≤2	high	12	11(91.7%)	1(8.3%)	
	low	19	0(0.0%)	19(100.0%)	<0.001
>2, ≤3	high	22	17(77.3%)	5(22.7%)	
	low	28	2(7.1%)	26 (92.9%)	<0.001
>3, ≤5	high	25	19 (76.0%)	6 (24.0%)	
	low	17	1 (5.9%)	16 (94.1%)	<0.001
>5, ≤7	high	7	4(57.1%)	3(42.9%)	
	low	5	0(0.0%)	5(100.0%)	0.038
>7	high	2	2(100.0%)	0(0.0%)	
	low	3	0(0.0%)	3 (100.0%)	0.025
Lymph node metastasis					
0	high	27	21 (77.8%)	6 (22.2%)	
	low	25	1 (4.0%)	24 (96.0%)	<0.001
1-3	high	17	13 (76.5%)	4 (23.5%)	
	low	25	1 (4.0%)	24 (96.0%)	<0.001
4-9	high	15	12 (80.0%)	3 (20.0%)	
	low	15	0 (0.0%)	15 (100.0%)	<0.001
≥10	high	5	4 (80.0%)	1 (20.0%)	
	low	7	1 (14.3%)	6(85.7%)	0.023
Lymph node invasion					
No	high	26	20 (76.9%)	6 (23.1%)	
	low	25	1 (4.0%)	24 (96.0%)	<0.001
Yes	high	38	30 (78.9%)	8 (21.1%)	
	low	47	2 (4.3%)	45 (95.7%)	<0.001

## DISCUSSION

Breast cancer is a significant public health problem in society today. Its pathogenesis involves a variety of gene expression abnormalities. The role of MAP2K4 has been reported to be inconsistent functions in different types of tumors, which can serve as either a tumor suppressor or an oncogene [13, 18, 19]. Contradictory research results have heightened interest in discovering the precise role, specific functions, and mechanisms of MAP2K4 in the development of breast cancer.

In this study, we started by overexpressing MAP2K4 in breast cancer cells to reveal the biologic functions of MAP2K4 in breast cancer and showed that MAP2K4 promoted cell proliferation, migration, and invasion *in vitro* and *in vivo*. Subsequently, the reverse effects were observed by silencing MAP2K4. These results verified that MAP2K4 plays an oncogenic role in breast cancer and promotes the occurrence and development of breast cancer, which is consistent with our previous study on breast cancer tissues [16]. However, the pathway by which MAP2K4 promotes breast cancer remained undefined.

Phosphoinositide 3-kinases (PI3Ks) affect many cellular biologic functions regarding intracellular signal transduction pathways and are one of the most common signaling pathways that are deregulated in cancer [20]. Growing evidence suggests that PI3K/AKT activation is vital to the induction of cell growth, metabolism, the EMT, and cancer stem cell (CSC) activities in tumor cells [21–23]. When PI3K is activated, it stimulates downstream AKT, and further modulates cell cycle and EMT-related gene expression, accelerating cell cycle progression, invasion, and metastasis [24, 25]. In this study, we first investigated the oncogenic role of MAP2K4 breast cancer, then we found that MAP2K4 overexpression upregulated p-PI3K and p-AKT. Furthermore, inhibiting p-PI3K expression using its specific inhibitor can reverse phenotypic changes in MAP2K4-overexpression breast cancer cells. Therefore we preliminarily concluded that MAP2K4 may activated the PI3K/AKT signaling pathway, upregulated the expression of cell cycle transforming factors (c-Myc, c-Jun, CCND1, and CDK2) and EMT signals (N-cadherin, Vimentin, and Slug), and meanwhile downregulated the expression of the inhibition factor E-cadherin. These data strongly demonstrated that MAP2K4 increases cell growth, migration, and invasion via the PI3K/AKT signaling pathway in breast cancer cells. These data strongly demonstrated that MAP2K4 increases cell growth, migration, and invasion via the PI3K/AKT signaling pathway in breast cancer cells. However, the specific molecular mechanism of how MAP2K4 activates the phosphorylated PI3K/AKT signaling pathway remains to be further studied.

Vimentin, as a classic EMT biomarker, is upregulated during the EMT in epithelial cells and induces mesenchymal phenotypes and motile behavior [26]. Vimentin has been shown to interact with a variety of proteins such as GlcNAc, p62, and other molecules, to promote invasion in multiple malignant tumor types [27, 28]. In this study, we used a co-immunoprecipitation and immunofluorescence to innovatively discover that MAP2K4 interacts with Vimentin. To confirm the specific effects of Vimentin in breast cancer, we subsequently inhibited Vimentin expression in MAP2K4-overexpressing MCF-7 and MDA-MB-231 cells and found that Vimentin suppression simultaneously reduced cell growth, the G1 to S cell-cycle transition as well as invasive and migratory abilities compared with the corresponding control cells.

In previous studies, the loss of MAP2K4 expression often indicated poor prognoses in human malignancies [29–31]. Concurrently, some statistical analyses of MAP2K4 expression and clinicopathologic features indicated that MAP2K4 could serve as a pro-oncogenic molecule [32]. Moreover, it has been reported that increased Vimentin expression was associated with poor prognoses in several different tumor types [33]. To explore the clinical prognostic value of MAP2K4 in breast cancer, we examined MAP2K4 and Vimentin expression levels in breast cancer tissues and analyzed the correlations between the expression of these markers and the clinicopathologic parameters.

In previous study**,** low MAP2K4 expression and positive Vimentin expression were reported to be associated with poor prognoses in endometrial carcinoma [34]. Furthermore, we also reported that increased MAP2K4 expression was a favorable prognostic factor in breast cancer [16]. To our knowledge, this study is the first investigation to measure MAP2K4 and Vimentin co-expression by immunohistochemical staining in breast cancer tissues and demonstrate a positive association between the levels of co-expression in breast cancer. In addition, we found that MAP2K4 and Vimentin expression that was markedly correlated with overall survival. Kaplan-Meier analysis indicated that patients with high expression of both MAP2K4 and Vimentin showed the worst prognoses compared with patients in the other three groups. These results indicated MAP2K4 and Vimentin co-expression to be a prognostic predictor of patient survival.

In summary, MAP2K4 activates the phosphorylated PI3K/AKT signaling pathway to activate downstream cycle-associated proteins and EMT signals, which promotes cell proliferation, migration, and invasion in breast cancer. Moreover, MAP2K4 interacts with Vimentin further accelerating cell proliferation, migration, and invasion, and co-expression of MAP2K4 and Vimentin was found to be an unfavorable factor in breast cancer prognosis. Therefore, we strongly speculate that MAP2K4 has an oncogenic role in breast cancer, which is at odds with a previous study [35]. Our study could provide new ideas for novel targeted therapies for breast cancer.

## MATERIALS AND METHODS

### Cell culture

Two breast cancer cell lines (MCF-7 and MDA-MB-231) were obtained from the Cell Bank of the Chinese Academy of Science (Shanghai, China) and were cultured in Dulbecco’s modified Eagle medium (DMEM) (Invitrogen) supplemented with 10% fetal bovine serum (FBS; Hyclone, Invitrogen). All cell lines were incubated in a 5% CO_2_ humidified chamber at 37°C.

### Lentivirus production and infection

Lentiviral particles carrying the MAP2K4 precursor were constructed by GeneChem (Shanghai, China). MCF-7 and MDA-MB-231 cells were infected with the lentiviral vector, and polyclonal cells with green fluorescent protein signals were selected for further experimentation using fluorescence-activated cell sorting (FACS) analysis.

### Small-interfering RNA (siRNA) knockdown of MAP2K4 in breast cancer cells

siRNAs for MAP2K4 were designed and synthesized by RiboBio Inc. (Guangzhou, China) Twenty-four hours before transfection, MCF-7 and MDA-MB-231 cells were plated into 6- or 96-well plates (Nest Biotech, China) and cultured to 30–50% confluence. siRNAs (si-MAP2K4-1 or si-MAP2K4-2) or negative control siRNAs (si-NC) were then transfected at a working concentration of 100nM using the Lipofectamine 2000 Transfection Reagent (Invitrogen, Carlsbad, CA, USA) according to the manufacturer’s protocol. MAP2K4 expression was confirmed by qRT-PCR and Western blot analysis.

### RNA extraction and qRT-PCR

Total RNA was extracted from the breast cancer cell lines using the Trizol Kit (Takara Bio, Inc., Shiga, Japan) according to the manufacturer’s instructions. GAPDH genes were used as internal gene controls. Cycling conditions were 95°C for 10min to activate DNA polymerase, followed by 45 cycles of 95°C for 15s, 60°C for 15s, and 72°C for 10s. Amplification product specificity was confirmed by melting curve analyses. Independent experiments were done in triplicate. Specific sense primers for MAP2K4 and GAPDH are shown as follows: MAP2K4 (Sense 5′-TCCCAATCCTACAGGAGTTCAA-3′, Antisense 5′- CCAGTGTTGTTCAGGGGAGA -3′), GAPDH (Sense5′- TGCACCACCAACTGCTTAGC -3′, Antisense5′- GGCATGGACTGTGGTCATGAG -3′)

### Western blot analysis

Total protein extracts were separated by 10% SDS‐PAGE and transferred onto PVDF membranes (Millipore, Bredford). Antibodies included MAP2K4, Vimentin,CCND1, c-JUN, c-Myc, AKT, pAKT (Ser473), PI3K, pPI3K (Tyr458), Slug, E-cadherin and N-cadherin were used in the Western blot analyses according to the manufacturer’s instructions. Detection was performed using the ECL Plus Western blotting detection reagents (Millipore, USA). The specific protein expression levels of the blots were normalized to GAPDH or β-actin.

### The cell proliferation assay

The breast cancer cells, MCF-7 and MDA-MB-231 (1,000/well or 2,000/well), were seeded into 96-well plates. For lentivirus-mediated MAP2K4 overexpression, the cells were incubated for 1, 2, 3, 4, 5, 6, or 7 days. For transient transfections with si-MAP2K4, cells were cultured for 1, 2, 3 or 4 days. Subsequently, 20ml of MTT (5mg/ml in PBS) (Sigma, St Louis, MO) solution was added to each well and incubated for 4h. Then, formazan crystals formed by viable cells were solubilized in 150ml dimethyl sulfoxide (Sigma, St Louis, MO) and the absorbance value (OD) was measured at 490nm. All the experiments were repeated at least three times.

### Flow cytometric analysis of the cell cycle

For the cell-cycle analysis, a total number of 5×10^6^ breast cancer cells were harvested after a 48h incubation and then washed with cold PBS. The cells were then fixed with 80% ice-cold ethanol at −20°C overnight. After incubation with PBS containing 10mg/ml propidium iodide and 0.5mg/ml RNase A for 15min at 37°C, fixed cells were washed with cold PBS three times. A FACS caliber flow cytometer (BD, Biosciences) was used to measure the DNA content of labeled cells.

### The 5-ethynyl-2'-deoxyuridine (EdU) incorporation assay

For the EdU incorporation assay, proliferating breast cancer cells were examined using a Cell-Light EdU Apollo 488 or 567 In Vitro Imaging Kit (RiboBio) according to the manufacturer’s protocol. Briefly, after incubation with 10mM EdU for 2h, breast cancer cells were fixed with 4% paraformaldehyde, permeabilized in 0.3% Triton X-100 and stained with Apollo fluorescent dyes. A total of 5mg/ml of DAPI was used to stain the cell nuclei for 10min. The number of EdU-positive cells was counted under a fluorescent microscope in five random fields. All assays were independently performed three times.

### The transwell invasion and migration assays

The invasion and migration assays were performed in a 24-well Transwell chamber with an 8μm pore-size filter membrane (Corning, Inc., New York, NY) coated with or without 50μl Matrigel (1:6 dilution; BD Biosciences). Cells in serum-free medium were seeded in the upper chamber, while conditioned medium with 20% FBS was added to the lower chamber. The chambers were incubated for 14h at 37°C. Non-migrated cells in the upper chamber were removed with cotton swabs, whereas migrated cells on the underside of the filter membrane were fixed in 4% paraformaldehyde and stained with Giemsa. The cell invasion assay procedure was identical to that of cell migration. The migrated/invaded cells were counted by light microscopy. The migration/invasion ability was measured by the mean cell number of five visual fields at a 200× magnification.

### In vivo tumorigenesis studies in nude mice

A total of 5×10^6^ logarithmically growing breast cancer cells (MCF-7) overexpressing MAP2K4 and their corresponding control cells were injected into the fourth pair of nude mice breast fat pads (BALB/C, nu/nu, 3–4 weeks-old, female).

The animals were fed an autoclaved laboratory rodent diet. On the 25^th^ day, the mice were euthanized, and tumor tissues were excised and weighed. All animal studies were conducted in accordance with the principles and procedures outlined in the Southern Medical University Guide for the Care and Use of Animals.

### Co-immunoprecipitation (Co-IP)

Co-IP was conducted using the Pierce Co-IP Kit (Thermos Scientific, USA) according to the manufacturer’s instructions. Total proteins were extracted and quantified. A total of 3000μg protein in 400μL supernatant was incubated with 10μg anti-MAP2K4, anti-Vimentin, or anti-IgG antibodies for 12 h at 4 °C. Beads were washed, eluted in a sample buffer, and boiled for 10 min at 100 °C. Immune complexes were subjected to Coomassie Brilliant Blue staining and Western blot analysis. Anti-IgG was used as a negative control.

### The confocal assay

MCF-7 cells were co-transfected with the MAP2K4 and Vimentin plasmids in a six-well plate. Cells were cultured overnight before they were fixed with 3.5% paraformaldehyde and permeabilized with 0.2% Triton X-100 at room temperature. The cells were incubated with anti-MAP2K4 and anti-Vimentin vimentin antibodies for 30–45 min at 37°C. After incubation for 30-45 min at 37 °C with a secondary antibody, coverslips were mounted onto the slides with a mounting solution containing 0.2mg/mL DAPI. Images were captured by laser scanning and confocal microscopy (ECLIPSE-Ti, Nikon).

### Immunohistochemical staining

Tissue sections from the in vivo experiments were used to detect Ki-67 and PCNA protein expression levels using immunohistochemistry. The indirect streptavidin-peroxidase method was used according to the manufacturer’s introduction. Immunohistochemically stained tissue sections were examined separately by two pathologists. The antibodies used were rabbit anti-PCNA (Cat. No.10205-2-AP, 1:30, Proteintech), anti-Ki67 (Cat. No. Ab16667, 1:100, Abcam).

Paraffin tissue arrays of breast cancer (HBre-Duc140Sur-01) was purchased from Shanghai Outdo Biotech (Shanghai Outdo Biotech Co., Shanghai, China). Tissue sections stained immunohistochemically for MAP2K4 and Vimentin were reviewed, and cytoplasmic staining scored separately by two pathologists blinded to the clinical parameters. The extent of staining, defined as the percentage of positively staining tumour cells in relation to the whole tissue area, was scored on a scale of 0-4 as follows: 0, <10%; 1, 10-25%; 2, 26-50%; 3, 50-75%; and 4, >75%. The staining intensity was scored as 0-3(Negative:0; Weak expression: 1; Positive expression:2; Strong expression:3; The sum of the staining intensity and staining extent scores was used as the final staining score for MAP2K4 and Vimentin(0-7). For statistical analysis, final staining scores of 0–5 and 6–7 were considered to respectively show low and high expression.

### Statistical analysis

Statistical analyses were performed with the SPSS 13.0 statistical software package (SPSS Inc. Chicago, IL, USA). Data are expressed as the mean ± SD from at least three independent experiments. Two-tailed Student’s t-test was used for comparisons between groups. Associations between MAP2K4 and Vimentin were analyzed using the Spearman’s correlation coefficient. Survival analysis was performed using the Kaplan–Meier method. All statistical tests were two-sided, and single, double, and triple asterisks indicate statistical significance(*P<0.05, **P<0.01 and ***P<0.001).
